# Potency of Hyperbaric Oxygen Therapy on Oral Candidiasis Diabetes Mellitus

**DOI:** 10.1055/s-0044-1779425

**Published:** 2024-03-31

**Authors:** Kristanti Parisihni, Dian Mulawarmanti, Kharinna Widowati

**Affiliations:** 1 Department of Oral Biology, Faculty of Dentistry, Universitas Hang Tuah, Surabaya, Indonesia; 2 Department of Oral Medicine, Faculty of Dentistry, Universitas Hang Tuah, Surabaya, Indonesia

**Keywords:** hyperbaric oxygen therapy, oral candidiasis, diabetes mellitus, lymphocytes, hyperglycemia

## Abstract

**Objectives** This study aimed to determine the potency of hyperbaric oxygen therapy (HBOT) to the blood glucose level, amount of lymphocytes, and the expression of antibody anti- *Candida* in oral candidiasis diabetes mellitus.

**Materials and Methods** Thirty Wistar rats were divided into five groups: K1 normal-control group, while K2, P1, P2, and P3 were diabetes induced by streptozotocin 50 mg/kg body weight. Oral candidiasis was induced by inoculation 0.1 mL *Candida albicans* ATCC 10321 on dorsum of the tongue. P1 was given nystatin oral suspension, P2 was treated by HBOT 2.4 ATA, 3 × 30 minutes each with a 5-minute interval, and P3 was treated by the combination of nystatin and HBOT. All the treatments were performed for 5 consecutive days. Fasting blood glucose level was examined on glucometer strips, lymphocyte was examined from complete blood count, and the expression of antibody anti- *Candida* was examined on immunohistochemistry staining

**Statistical Analysis** Data analyzed by a one-way analysis of variance and least significant difference test with the result of *p* -value less than 0.05 was considered to be statistically signiﬁcant.

**Results** HBOT reduced blood glucose level ( *p* > 0.05) increased the amount of lymphocyte ( *p* < 0.05). All treatments reduced the expression of antibody anti- *Candida* ( *p* < 0.05) with the best result on combination of HBOT with nystatin.

**Conclusion** HBOT decreased the blood glucose level, increased the amount of lymphocytes, and reduced the expression of antibody anti- *Candida* in oral candidiasis diabetes mellitus.

## Introduction

Diabetes mellitus (DM) is a systemic disease refers to a diverse range of clinical and genetic metabolic conditions characterized by elevated blood glucose level. 1 Diabetes imposes a significant strain on healthcare system, with data from the International Diabetes Federation suggesting that 537 million people worldwide were living with diabetes in 2021. Hyperglycemia condition leads to damages to various organs of the body and also manifest in high prevalence of oral complications such as dental caries, xerostomia, periodontal disease, sensory disorders, taste problems, salivary gland dysfunction, and oral infections. 2

DM predisposes individuals to fungal infections, including oral candidiasis due to immunosuppressive condition. Numerous immune alterations have been described in DM showing some changes in polymorphonuclear cells, monocytes, and lymphocytes. Multiple factors are associated with higher *Candida* sp susceptibility in DM regarding localized or systemic infection. These factors include the adherence of yeast to epithelial cell surface, diminished salivary low, deterioration of microvascular function, and reduced neutrophils candidacidal activity. 3 Increasing evidences stated that lymphocyte contributes significantly to the host defense against *Aspergillus* and *Candida* infection. 4 The decrease in lymphocyte count results in a weakened immune defense against fungi in the mucosa and epidermis, leading to an increased number of *Candida albicans* and the development of oral candidiasis.

Current treatments for oral candidiasis in DM involve using topical antifungal medications like nystatin or certain azoles such as fluconazole. There is evidence suggesting that *Candida* species, especially *Candida albicans* , have the ability to develop resistance to antifungal agents, leading to a reduction in the plasma availability of fluconazole. Yet, there is limited data available on its resistance to commonly employed antifungal agents for oral candidiasis. Metabolic decompensation in DM resulted in changes of the saliva quality and composition, due to increasing glucose level in saliva. 5

Hyperglycemia plays a crucial role in influencing the emergence of DM complication. Oral candidiasis can be prevented by maintaining oral hygiene and controlling blood sugar levels through proper medication. 6 However, since impaired immune response also plays important role in pathogenic mechanisms contribute to DM complication, a novel strategic therapy affected those two mechanism of action should be considered to develop.

Hyperbaric oxygen therapy (HBOT) is a treatment that administers pure oxygen at 100% concentration under a pressure ranging from 2 to 3 atmosphere absolute (ATA) within hyperbaric chamber. The mechanism of HBOT is to enhance oxygen levels in body tissue leads to faster wound healing, inflammation reduction, and elimination of anaerobic bacteria. Emerging evidence indicated that HBOT may lead to a decrease in blood sugar level among individuals with DM. 7

Study by Mulawarmanti et al demonstrated that the administration of HBOT to diabetic rats induced with periodontitis resulted in an increase in osteoprotegerin production by osteoblasts and other cells such as lymphocytes in diabetic rats treated with HBOT. 8 Other than DM condition, it has been stated that the administration of HBOT at a pressure of 2.4 ATA for three sessions of 30 minutes per day with a 5-minute interval of breathing normal air, given for five consecutive days, could increase the number of lymphocytes in the blood during immunosuppressive conditions, thereby suppressing oral candidiasis infection caused by *Candida* *albicans* . 9

HBOT showed promising result as an approach to overcome oral complication in DM. This study aimed to determine the potency of HBOT to the blood glucose level, amount of lymphocytes, and the expression of antibody anti- *Candida* in oral candidiasis DM.

## Materials and Methods

The study is experimental research with post-test only control group design that received approval from the Ethical Clearance for Health Research Committee, Faculty of Dentistry, Hang Tuah University, Surabaya, Indonesia No: EC/005/KEPK-FKGUHT/V/2023.

### Diabetes Mellitus Animal Model Induction

Thirty male *Rattus norvegicus* Wistar rats weighing between 150 and 200 g were randomly divided into five groups: K1 is normal-control group, while K2, P1, P2, and P3 are animal model of oral candidiasis DM. Diabetes condition was induced by streptozotocin (Bioworld, Dublin) 50 mg/kg body weight intra peritoneal, single dose. 10 Diabetic condition is stated and characterized by the high level of blood glucose of 220 mg/dL or more. 11

### Oral Candidiasis Induction

Right after the diabetic condition established then begun the induction of oral candidiasis. Tetracycline was administered at a dose of 1 mg per rat via a rat oral gavage to control the oral cavity condition prior to fungal induction. Oral candidiasis was induced by inoculation 0.1 mL 10 ^9^ Mc Farland *Candida albicans* ATCC 10321 swabbed on to dorsum of the tongue surface every 2 days and repeated for three times. After the induction of *Candida albicans* , a swab was taken from the tongue using a sterile cotton swab and cultured on chromogenic agar media to confirm the presence of *Candida albicans* fungal infection. 9

### HBOT and Nystatin Treatment

Treatment was administered on group P1, P2, and P3. Group P1 was given nystatin oral suspension topically on dorsum of tongue, once a day. Group P2 was treated by HBOT 2.4 ATA, 3 × 30 minutes each with a 5-minute interval in hyperbaric animal chamber. 8 9 Group P3 was treated by the combination of nystatin and HBOT as mentioned above. All treatments were performed for 5 consecutive days.

### Sample Collection and Examinations

Fasting blood glucose level was examined on glucometer strips (Bioptic Inc., Taipei) three times: before and after 5 days of streptozotocin induction, and the day prior to termination. Blood sample was collected for total blood count examination for lymphocyte. Sample from tongue slide section performed immunohistochemistry staining (SantaCruz Biotech, Texas) and the expression of antibody anti- *Candida* on the macrophage was examined under the microscope (Olympus, Japan) at 400x magnification. The data was analyzed by a one-way ANOVA and least significant difference test on which a result of *p* -value less than 0.05 was considered statistically signiﬁcant.

## Results

HBOT has been proved to have the impact on blood glucose level, amount of lymphocytes, and the expression of antibody anti- *Candida* in oral candidiasis DM as shown in Table 1

**Table 1:** Mean and standard deviation of blood glucose level, lymphocyte count, antibody anti-Candida expression in all group

Group	Blood glucose level	Lymphocyte count	Antibody anti- *Candida* expression
K 1	113 ± 13.5 a	73,5 ± 10.1 a	0 ± 0 a
K 2	595 ± 9.8 a	61 ± 12.4 a	12.2 ± 2.2 a
P 1	570 ± 41.8 a	62 ± 15.3 a	6 ± 1.4 a
P 2	446 ± 214.6 a	71 ± 10.1 a	7.2 ± 0.9 a
P 3	447 ± 173.1 a	74 ± 9.8 a	2.3 ± 0.5 a

a Difference between the groups with significance level of 5% ( *p* < 0.05).

**Fig. 1:**
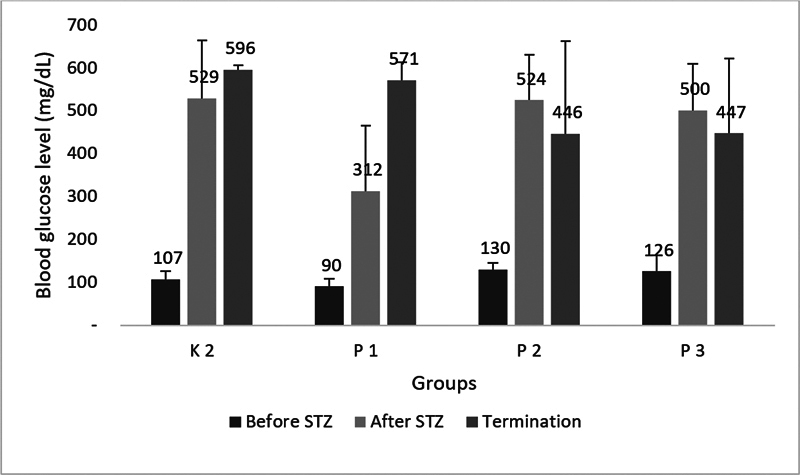
Mean of blood glucose level (mg/dL), before streptozotocin induction, after streptozotocin induction, and at the termination time in oral candidiasis diabetes mellitus rats.

Streptozotocin induction resulted in DM condition indicated by elevated blood glucose level after Streptozotocin (STZ) administration ( Fig. 1 ). Hyperglycemia condition raised higher in oral candidiasis DM untreated group K2 and nystatin oral suspension treatment. While treatment with HBOT both in P2 dan P3 group showed reduced hyperglycemia but still on the DM range criteria blood glucose level ( *p* > 0.05).

Lymphocyte count was decreased on all groups with oral candidiasis DM compared with normal ( *p* < 0.05). HBOT both alone (P2) or in combination with nystatin (P3) increased lymphocyte count ( *p* < 0.05).

**Fig. 2:**
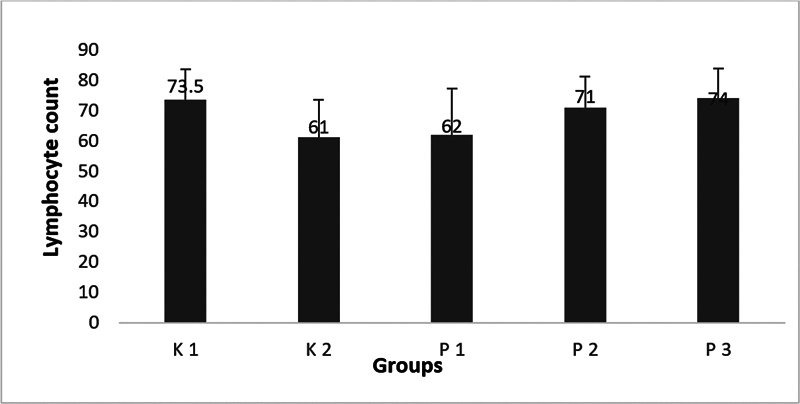
Mean of lymphocyte count in all groups.

Since *Candida* sp is not normal flora of the rats, antibody anti- *Candida* was not obtained in normal group K1, but can be observed in all groups with oral candidiasis DM ( Fig. 2 ).

**Fig. 3:**
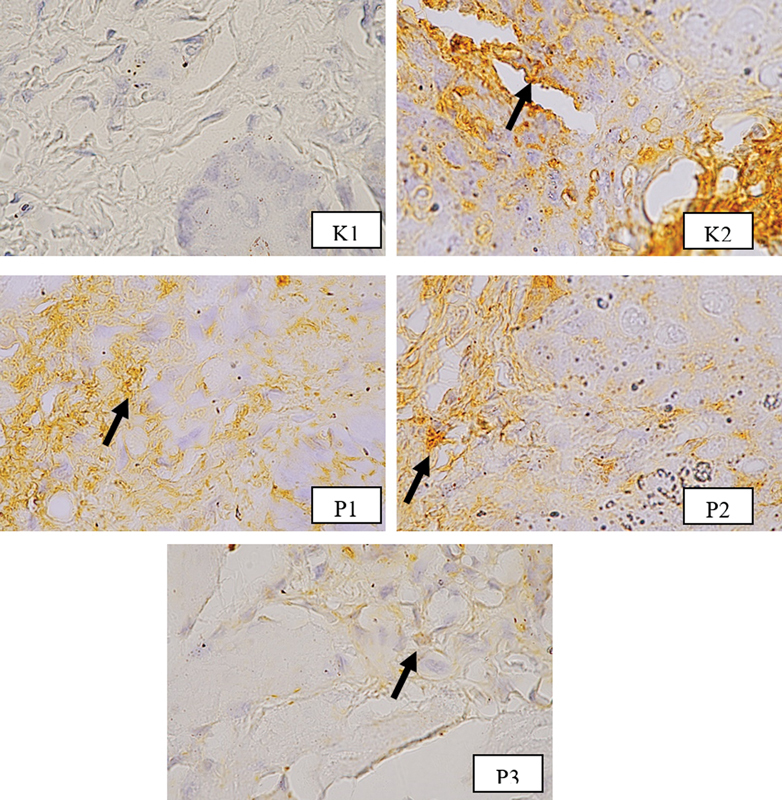
Immunohistochemistry staining on tongue slide of the rats. Healthy normal subject showed no expression of antibody anti- *Candida* (K1), The black arrows indicate the expression of antibody anti-Candida on untreated oral candidiasis diabetes mellitus (K2) and in all treatment groups (P1, P2, P3) (at 400x magnification).

**Fig. 4:**
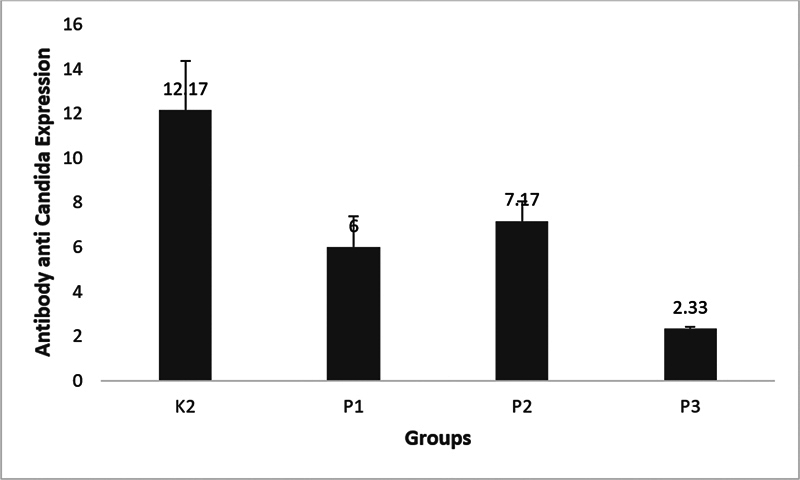
Mean expression of antibody anti- *Candida* in all groups of oral candidiasis diabetes mellitus.

Expression of antibody anti- *Candida* on rat tongue is shown in Fig. 3 . Treatment with nystatin, HBOT, and combination of both reduced the expression of antibody anti- *Candida* ( *p* < 0.05) as shown in Fig. 4. The best result of reduction was on combination of HBOT with nystatin.

## Discussion

Oral candidiasis is a disease caused by the pathogenic fungus *Candida* *albicans* . Oral candidiasis is influenced by several predisposing factors such as xerostomia, local trauma, malnutrition, long-term use of antibiotics, HIV, and DM. 12 DM can cause oral candidiasis because conditions of high blood sugar levels lead to decrease in the ability of phagocytosis and polymorphonuclear in killing foreign bodies. Therefore, the epithelial cells in the mouth experience increased adhesion to *Candida* and there is an increase in overgrowth of *Candida* . 8

In this study, the DM condition has been achieved by the administration of streptozotocin. The diabetic agent induction was able to inhibit the pancreatic Langerhans β cells for the production of the hormone insulin by producing free radicals that are very reactive so that they damage cell membranes, proteins, and deoxyribonucleic acid 10 presented in stable hyperglycemia condition until the end of termination ( Fig. 1 )

Group treated with only nystatin showed no reduction in blood glucose level. Nystatin is common antifungal therapy and has its effectiveness as treatment in oral candidiasis 13 but in the condition of DM it does not affect the hyperglycemia control regarding its mechanism of action solely in antifungal property. HBOT both alone and combination with nystatin proved to reduce the blood glucose level. The decrease in blood glucose levels resulting from HBOT seems to be partially due to improved insulin sensitivity rather than increased insulin secretion. 7 14

However, the decrease in hyperglycemia was not notably significant, as the blood glucose levels remained high compared to normal. This outcome might be subject to greater impact by further research, particularly by exploring treatment durations longer than the five days performed in this study. Liu et al conducted a study which proved that blood sugar could decrease if HBOT was performed for 7 days, so further research is needed on the effect of HBOT on blood sugar, 14 while another study stated that HBOT had the impact on glycaemia condition in 10 sessions or more. 7

Lymphocytes count in untreated oral candidiasis DM group decreased in compared with normal healthy group ( Fig. 2 ). Conditions of high blood sugar levels (hyperglycemia) lead to a rise in advanced glycation end-products (AGEs), triggering heightened oxidative stress and a reduction in the distribution of lymphocytes. An increase in oxidative stress causes an increase in proinflammatory cytokines and a decrease in anti-inflammatory cytokines. 15 16 17 The decrease in the number of lymphocytes causes the body's defense against microorganisms such as fungi to decrease so that the growth of fungal colonies increases. In addition, vascularization of the oral cavity will also be disrupted and followed by increased adhesion to fungi in the oral cavity, causing manifestations of oral candidiasis in the oral cavity. 6 16

Diabetics experience prolonged inflammation, leading to delayed healing. This delay is a consequence of factors such as impaired neutrophil recruitment, compromised macrophage function, continuous release of proinflammatory cytokines, impaired keratinocytes, and hindered fibroblast proliferation. Macrophages, in particular, exhibit altered functions, displaying an increased inflammatory profile and reduced phagocytic capacity. The delayed recruitment of neutrophils is linked to the elevated deposition of AGEs, which directly inhibit the chemotactic activity of neutrophils. Additionally, the activation of reactive oxygen species (ROS) induces lymphocyte apoptosis, further hindering the healing cascade. 18 The immunomodulatory properties of HBOT could potentially extend to the regulation of inflammatory responses and related complications. Notably, HBOT is associated with significant immunomodulatory effects, including changes in the CD4 + :CD8+ ratio, decreased lymphocyte proliferation, and the activation of neutrophils with migration to hyperoxic regions. 19 HBOT has been shown to possess anti-inflammatory effects by decreasing the production of proinflammatory cytokines. Chronic inflammation is known to be a contributing factor to *Candida* overgrowth, and the anti-inflammatory properties of HBOT may play a role in managing fungal infections. 20

HBOT is a therapy performed by providing 100% pure oxygen in a high-pressure room of more than 1 ATA. 15 Previous studies have shown that administration of HBOT at a pressure of 2.4 ATA 3 × 30 minutes/day with 5-minute intervals of inhaling plain air given for five consecutive days can increase the number of lymphocytes in the blood in immunosuppressed conditions so as to suppress oral candidiasis infection. 6

The results also showed a significant increase in the number of lymphocytes between the group of mice without HBOT and the group of mice that were given HBOT. This shows that exposure to high pressure oxygen on HBOT resulted in increase in IFN-γ, inducible nitric oxide synthase, and vascular endothelial growth factor. IFN-γ induces an increase in TH-1, which influences B lymphocyte cell proliferation. 9 HBOT at a pressure of 2.4 ATA was able to produce antioxidants and reduce cell damage, especially blood cells. This was also followed by an increase in the number of erythrocytes, lymphocytes, and thrombocytes. HBOT could also increase oxygen in the cells and will be followed by an increase in ROS. Increased ROS functions as an antioxidant signaling molecule in the process of vascularization and healing. 21

The specific antibody response to Candida proteins has reported to be induced in both immunocompromised and immunocompetent patients. Detection of *Candida* antigens and antibodies has been performed to have practical clinical value specially in cases of invasive candidiasis as the development of nonculture-based laboratory method. 22 Evaluation on the expression of antibody anti- *Candida* in this study reflected the amount of *Candida* in oral candidiasis DM of the rats.

Result showed no antibody anti- *Candida* expressed in healthy normal rat in K1 group, but appeared in all rest of the groups with oral candidiasis DM ( Fig. 3 ). *Candida* sp could be isolated from human oral cavity and the most common cause of fungal disease particularly in oral and vaginal mucosae. But in animal model the condition of *Candida* acquisition is not quite the same. Rats may carry *Candida albicans* in their oral cavity, although the prevalence of this occurrence is less compared to humans. It's worth noting that *Candida* is not a common resident of the oral microbiome in mice. 23

The expression of antibody anti- *Candida* raised highly in untreated oral candidiasis DM ( Fig. 4 ). There are numerous factors that contribute to an increased susceptibility to *Candida* infections in patients with DM and promote the colonization of *Candida* in the host such as adherence of yeast to epithelial cell surfaces, elevated levels of glucose in saliva, reduced saliva production, microvascular damage, and weakened *Candida* -killing ability of neutrophils. These conditions become particularly concerning when there is excess glucose, and significantly impact the balance between the host and *Candida* from commensal to pathogen resulted in oral candidiasis. 4 6

Treatments with nystatin, HBOT, and its combination reduced the expression of antibody anti- *Candida* ( Fig. 4 ). ROS produced by HBOT could activate signal cascades so that they can provide beneficial effects on lymphocyte cells. The formation of ROS also stimulates lipid peroxidation. Lipid peroxidation destroys cell membrane lipids in fungi that can lead to dysfunction. Damage to the plasma membrane can inhibit the growth of fungi that is characterized by reduced *Candida albicans* colonies. 24 25

It has been showed that hyperbaric oxygen has fungicidal effects against various fungi and yeasts. While it is not considered a standalone treatment, HBOT may complement conventional antifungal therapies. By creating an environment less favorable for *Candida* growth, HBOT can potentially enhance the efficacy of antifungal medications. Furthermore, there is evidence suggesting that HBOT, when combined with amphotericin B, can inhibit the growth of *Candida albicans* . This underscores the potential for synergistic effects between HBOT and antifungal agents in addressing fungal infections. 26

The administration of HBOT increased the number of lymphocytes in Wistar rats so that *Candida albicans* infection could be suppressed and the Wistar rats began to heal back to normal. This aligns with previous research by Pargaputri and Andriani stated that hyperbaric oxygen therapy (HBOT) at a pressure of 2.4 ATA, lasting for 3 sessions of 30 minutes each, with 5-minute intervals of breathing ordinary air, for a continuous period of 5 days, resulted in the increasing number of blood lymphocytes count. Immunosuppressed oral candidiasis Wistar rats and HBOT have been shown to be able to eliminate *Candida albicans* infection that causes oral candidiasis. 9 HBOT increased oxygen levels in the tissues so that oxidative stress would decrease, and could also enhance the function of immune cells so that the growth of fungal spores and fungal mycelium can inhibited. 24 27

An increase in the number of lymphocytes will be followed by an increase in antibodies that function to protect the body from foreign bodies, bacteria, and fungi. Lymphocytes release cytokines that can enhance the cellular immune response and modulate the antifungal activity of polymorphonuclear leukocytes and macrophages. Based on research conducted by Forsyth and Matthew, it is demonstrated that the proliferation of Candida albicans can be impeded by lymphocytes. This inhibition occurs through direct interactions with Candida albicans hyphae, involving intricate molecular mechanisms. Notably, IL-12 activated CD8þ lymphocytes play a role in directly hindering fungal growth, IL-12 activated NK cells release IFN-γ directly, and the SD11b/CD18 molecule serves as the primary structure in the attachment of lymphocytes to the fungal hypha. 25 27 Lymphocytes serve as the host's primary defense against fungal infections of the mucosal surfaces and epidermis. An increase in the number of lymphocytes may represent the first step in producing an antifungal effect against *Candida albicans* . 9

In this study, administration of HBOT 2.4 ATA 3 × 30 minutes with 5 minutes intervals inhaling ordinary air and performed for 5 consecutive days had a great effect on increasing the number of lymphocytes in diabetic rats so that they could suppress the growth of *Candida albicans* fungi and accelerate the healing of oral candidiasis. In concordance with Dhingra et al, HBOT could inhibit fungal growth on the 5th day of therapy. 28 Further research on optimizing the duration of the therapy and combination with antifungal agent might reveal the more potency of HBOT in oral candidiasis DM.

## Conclusion

HBOT decreased the blood glucose level, increased the amount of lymphocytes, and reduced the expression of antibody anti- *Candida* in oral candidiasis DM.
